# American Institute of Homeopathy

**Published:** 1897-04

**Authors:** A. R. Wright

**Affiliations:** Chairman of Local Committee, 414 Elmwood Avenue, Buffalo, N. Y.


### AMERICAN INSTITUTE OF HOMEOPATHY.

(Local Committee of Arrangements, Buffalo Meeting, 1897.)

EDITOR OF THE HOMEOPATHIC PHYSICIAN:—The local committee of arrangements for the next meeting of the

American Institute of Homeopathy, which meets in Buffalo, New York, June 23d to 30th, 1897, have made much progress with their preparations.

Buffalo being situated at a point near the middle west and readily accessible by numerous lines of railway east and west, presents a favorable opportunity for a large attendance of the profession.

The local committee have chosen Unity Hall for the meetings of the Institute. This hall, with its numerous connecting rooms and large auditorium, furnishes most admirable facilities for the work of the Institute, including accommodations for its numerous sectional meetings and committee rooms. It is located on Delaware Avenue, the chief residence street of the city, and within two or three squares of the leading hotels.

Ample hotel accommodations will be at hand. The Iroquois Hotel, which will be the official headquarters, $4.00 per day; the Tifft House, $2.50 to $4.00 per day; the Genesee, $2.50 per day upwards; the Fillmore, $2.50 per day; the Niagara, $3.00 and upwards per day; the Ontario, $2.50 and $3.00 per day; the Trubee, $2.50 per day and upwards.

The climate of Buffalo in June is very fine, and every opportunity can be given for enjoyment of the members during leisure hours. With something over 200 miles of asphalt pavement, those who ride wheels can luxuriate to their heart's content. The committee urge those who have wheels to bring them with them; they will be cared for by competent persons at Unity Hall, where, also, those who wish to hire wheels may do so at moderate rates; the whole under the arrangement and control of the committee.

Buffalo's elaborate system of public parks, which, with their connecting boulevards, nearly encircle the city, will prove a source of great interest to visitors. The botanical gardens at South Park are already attracting wide attention throughout the country.

In the Free Library Building, facing Lafayette Park, will be found a large free library conducted by the city; also a large collection of the Society of Natural Science; also that of the Historical Society and the Academy of Fine Arts.

Among the famous attractions of Buffalo are its mighty grain elevators, which handle the immense commerce of the great lakes, en route to the sea-port towns.

The office buildings will prove an attraction to many; the one called the Ellicott Square, covering a whole city block, is acknowledged to be the largest building in the world.

The electric street railways compose a safe, perfect system of travel, and furnish the first example of the long-distance electric transmission, the power being generated at Niagara Falls, twenty-two miles away.

An endless variety of excursions will be provided, by steam and electric railroad, to many interesting points, including Niagara Falls, which is close at hand; also by boat on Lake Erie and on Niagara River.

The trip to Niagara Falls will prove of surpassing interest to all visitors, and can be made by steam train in forty minutes, or by electric railroad.

The new and fascinating ride through the gorge of Niagara River from Niagara Falls to Lewiston by electric railroad, will prove a chief feature. It is called the "Gorge Route," and is a fascinating though perfectly safe excursion, the cars running low down in the gorge within a few feet of the water's edge.

The great power-house at Niagara Falls, where, by the aid of the current of the Niagara River, thousands and thousands of horse-power of electric energy are generated for commercial purposes.

The power-house will prove a great attraction to visitors.

Every effort will be made by the local committee for the comfort and entertainment of the members and guests of the Institute and their families; but no entertainments or excursions will be planned which will interfere with the more serious work of the Institute.

JOSEPH T. COOK, Per C. L. M., *Secretary Local Committee,*
636 Delaware Avenue, Buffalo, N. Y.
By order of DR. A. R. WRIGHT,
*Chairman of Local Committee,*
414 Elmwood Avenue, Buffalo, N. Y.
Buffalo, N. Y., March 13th, 1897.

---

